# Editorial: Vascular malformations and thrombosis

**DOI:** 10.3389/fmed.2023.1196902

**Published:** 2023-06-27

**Authors:** Pierpaolo Di Micco, Antoni Riera-Mestre, Egidio Imbalzano

**Affiliations:** ^1^Department of Medicine, Presidio Ospedaliero Santa Maria delle Grazie, Pozzuoli, Italy; ^2^Internal Medicine Department, Hospital Universitari de Bellvitge, Barcelona, Spain; ^3^Department of Medicine, Bellvitge Biomedical Research Institute (IDIBELL), L'Hospitalet de Llobregat, Spain

**Keywords:** vascular malformations, thrombosis, umbilical aneurysm, thrombocythemia, sclerotherapy, jugular vein agenesis, oral anticoagulants

Thrombotic disorders appear as acute diseases of arterial or venous vessels and represent the leading cause of morbidity and mortality in western countries. Risk factors differ among arterial or venous thrombosis, including molecular risk factors and clinical predisposing diseases. Together with predisposing diseases, congenital or acquired vascular malformations may also be associated with the occurrence of thrombosis or bleeding. Usually, thrombotic events of vascular malformations may appear as symptomatic disease, while bleedings are frequent in the case of vascular malformations such as aneurysms or arterio-venous fistulae. Furthermore, vascular malformations may be found in small or large vessels. From a clinical point of view, the most common difficulty for the management of vascular malformation is the absence of guidelines or best clinical practice. For this reason, and because patients with rare vascular malformations are not included in general guidelines, clinical evidence and experience in treating thrombotic or hemorrhagic complications in these patients are scarce, and further data regarding pathophysiological mechanisms and/or clinical outcomes are encouraged.

In this Frontiers Research Topic, several case reports described the occurrence of thrombotic events or bleeding in carriers of rare vascular malformations (Oualiken et al.; Li et al.; Zhou et al.). Furthermore, when a vascular malformation is identified before complication, a preventive strategy should be evaluated (Puhr-Westerheide et al.), as reported in the case of associated pregnancy (Di Micco et al.) or in the case of malformation of the face (Schmidt et al.). In other situations, such as acquired thrombophilia due to the presence of antiphospholipid syndrome, the clinical presentation may differ because antiphospholipid syndrome is a prothrombotic molecular abnormality that may simultaneously affect arterial and venous vessels in the case of vascular malformation (Jacintho et al.). On the other hand, a definite prothrombotic abnormality that can state the risk of thrombosis or bleeding of vascular malformations has not identified. Prothrombotic endothelial abnormalities may characterize clotting abnormalities, with a trend toward thrombosis in other prothrombotic diseases such as COVID-19 or Beçhet disease (Ma et al.; Thangaraju et al.). Individual lifestyle is also important, and the protective role of environmental food should be investigated in-depth in the next number of years, in particular, in selected cohorts of patients that seem to have a favorable genetic predisposition (Jia et al.).

Little is known concerning prophylaxis or therapeutic therapies regarding vascular malformations. Di Micco et al. reported successful prophylaxis using enoxaparin in a pregnant woman with jugular agenesis, while RIETE investigators described the clinical characteristics of patients treated with DOACs after a venous thromboembolism (VTE) event (Lorenzo et al.). Molecular abnormalities that may explain drug resistance in prothrombotic diseases as essential thrombocytopenia have been reported by Yang et al.. Furthermore, the role of prophylaxis in patients with vascular malformation has been tested, with positive results obtained, in particular, the quality of life in patients treated with sirolimus (Harbers et al.).

All the articles included in this Research Topic will help clinicians dealing with patients with rare vascular malformations, improving the management of these complex situations.

## Author contributions

All authors listed have made a substantial, direct, and intellectual contribution to the work and approved it for publication.

